# From chemical control to ecological resilience: Biological control and integrated pest management in arid agriculture

**DOI:** 10.1016/j.isci.2026.117334

**Published:** 2026-09-01

**Authors:** Seham M. Al Raish

**Affiliations:** 1 Department of Biology, College of Science, United Arab Emirates University, Al Ain, Abu Dhabi 15551, United Arab Emirates

**Keywords:** United Arab Emirates, agriculture, biological control, integrated pest management, pesticides, date palm, arid agroecosystems, food security

## Abstract

Agriculture in the United Arab Emirates supports food system resilience but operates under extreme heat, water scarcity, salinity, and persistent pest pressure. This systematized narrative review evaluates biological control and integrated pest management (IPM) as alternatives to intensive pesticide use, focusing on date palm, vegetables, and protected cultivation. Evidence indicates that routine pesticide dependence can accelerate resistance, disrupt beneficial organisms, increase occupational and residue risks, and threaten soil and water quality. UAE and Gulf studies show that pheromone monitoring, microbial agents, semiochemicals, sanitation, selective pesticides, and precision technologies can support more sustainable control, particularly against red palm weevil. However, variable field performance, limited local validation, farmer perceptions, advisory gaps, registration barriers, and weak incentives constrain adoption. A staged transition centered on monitoring, locally tested biological tools, stronger extension services, selective interventions, and aligned policy support offers the most credible and practical pathway toward resilient crop protection in arid UAE agriculture.

## Graphical abstract

**Figure:**
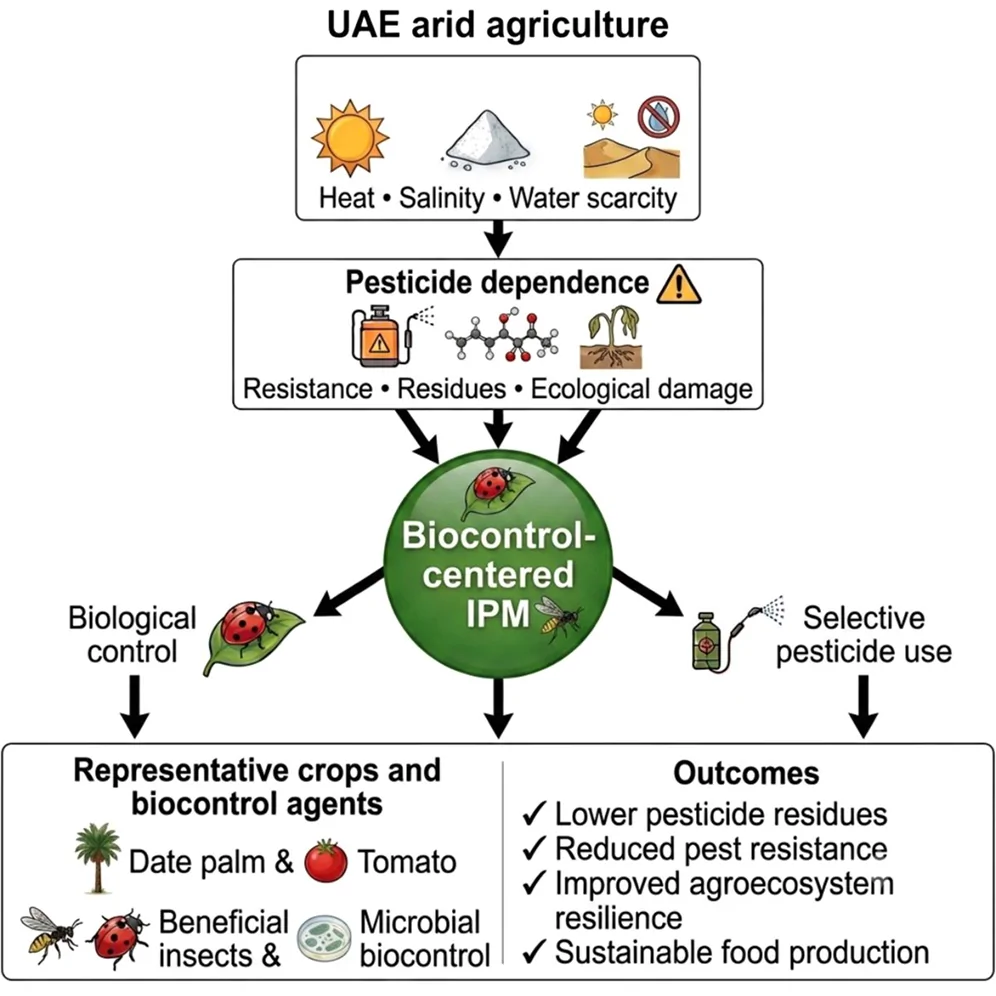

## Teaser

Agricultural science; Biocontrol in food

## Introduction

The UAE represents one of the most challenging environments in which to pursue sustainable crop production. High temperatures, low rainfall, limited arable land, groundwater salinity, and heavy dependence on external food supply create a structural tension between the desire to strengthen domestic production and the environmental limits of arid agriculture. For that reason, agricultural sustainability in the UAE cannot be understood only through output volumes. It must be interpreted in terms of resilience, strategic self-provisioning, and the capacity of agricultural systems to continue functioning under ecological stress. This framing is especially important for pest management, because crop protection decisions can either support or undermine long-term system resilience.^1^^,^^2^^,^^3^^,^^4^

The literature assembled for this review shows that pest management in the UAE sits at the intersection of food security, environmental stewardship, and public health.^5^^,^^6^^,^^7^^,^^8^^,^^9^ Domestic production is strategically important because the country remains highly exposed to supply-chain disruption, while major crops such as date palm, vegetables, and protected-cultivation horticulture are vulnerable to both pests and climatic stress.^5^^,^^6^ In such settings, chemical pesticides often appear attractive because they provide visible and rapid suppression. Yet the same body of literature also demonstrates that prolonged reliance on chemical control can create resistance, residues, biodiversity losses, occupational exposure, and contamination risks, all of which are amplified in fragile arid ecosystems.^10^^,^^11^^,^^12^^,^^13^^,^^14^^,^^15^^,^^16^^,^^17^^,^^18^^,^^19^

The evidence base is intentionally broader than a UAE-only agronomy review and links local crop-protection problems to the wider science of sustainable pest management. It spans UAE food-security and production-system studies,^5^^,^^6^^,^^20^^,^^21^ crop and date-palm resilience studies,^15^^,^^17^^,^^18^ UAE/Gulf pest-management evidence centered on red palm weevil and related threats,^12^^,^^15^^,^^16^^,^^19^^,^^22^ arid-zone soil, water, and climate constraints.^23^^,^^24^^,^^25^^,^^26^^,^^27^ Pesticide-use and pesticide-risk syntheses.^8^^,^^9^^,^^28^^,^^29^ Biological-control and microbial-input studies,^30^^,^^31^^,^^32^^,^^33^^,^^34^^,^^35^^,^^36^ IPM framework papers.^9^^,^^37^^,^^38^^,^^39^^,^^40^^,^^41^ and farmer-adoption or KAP-oriented studies that help interpret implementation barriers.^42^^,^^43^^,^^44^^,^^45^ The thematic classification of the curated evidence base is presented in Tables S1–S8.

This review provides an integrated assessment that moves beyond conventional discussions of pest-control efficacy to examine pest management in the UAE as a multi-scalar sustainability issue spanning national food security, farm-level productivity, environmental integrity, and occupational and consumer health. It combines UAE-specific evidence with broader arid-region scholarship to develop a context-sensitive synthesis of why chemical-dependent control remains entrenched and how biologically based and integrated approaches can support a transition toward more resilient agricultural systems.

## Review methodology

### Review design and scope

This article used a systematized narrative review design to synthesize heterogeneous evidence concerning chemical pesticide dependence, biological control, integrated pest management (IPM), and agricultural sustainability in the United Arab Emirates (UAE) and comparable arid and semi-arid production systems. A narrative synthesis was selected because the available evidence included empirical studies, review articles, modeling studies, field reports, institutional reports, and studies examining farmer knowledge, attitudes, practices, and technology adoption. The diversity of study designs, analytical objectives, and reported outcomes did not support quantitative pooling or meta-analysis. The review therefore followed a structured process of source cleaning, relevance screening, thematic organization, evidence appraisal, and critical narrative synthesis.

### Source corpus and eligibility criteria

The source corpus was drawn from a curated literature matrix assembled for this review. Sources were eligible when they contributed directly to at least one of six analytical domains: (1) the strategic importance of UAE agriculture and domestic food production; (2) climatic, hydrological, and soil-related constraints affecting agriculture in arid environments; (3) pest, disease, and weed pressures affecting major UAE crops, particularly date palm, vegetables, and protected-cultivation systems; (4) environmental, food-safety, occupational-health, and public-health consequences associated with chemical pesticide use; (5) biological control, microbial agents, semiochemicals, monitoring technologies, precision tools, and IPM; and (6) farmer adoption, extension, regulation, governance, and implementation barriers affecting sustainable pest-management transitions.

UAE-specific studies were prioritized because of their direct contextual relevance. Evidence from the Gulf region and other arid and semi-arid agricultural systems was retained when it offered biological mechanisms, management principles, technologies, or implementation lessons relevant to UAE conditions. Sources were excluded when they were duplicate or near-duplicate records, clearly outside the scope of the review, or did not materially contribute to the review question.

### Screening and thematic organization

The evidence-screening process was conducted in sequential stages. Duplicate and near-duplicate records were first removed. The remaining sources were then assessed for their relevance to the review question and the predefined eligibility domains. Eligible sources were organized into six thematic clusters: food-security context; arid production constraints; pest and crop vulnerabilities; pesticide dependence and associated risks; biological-control and IPM approaches; and adoption, extension, and governance barriers. Following removal of duplicate and near-duplicate records and assessment against the review objectives, eligible sources were retained in the curated evidence corpus and organized into six thematic domains for narrative synthesis.

**Figure 1:**
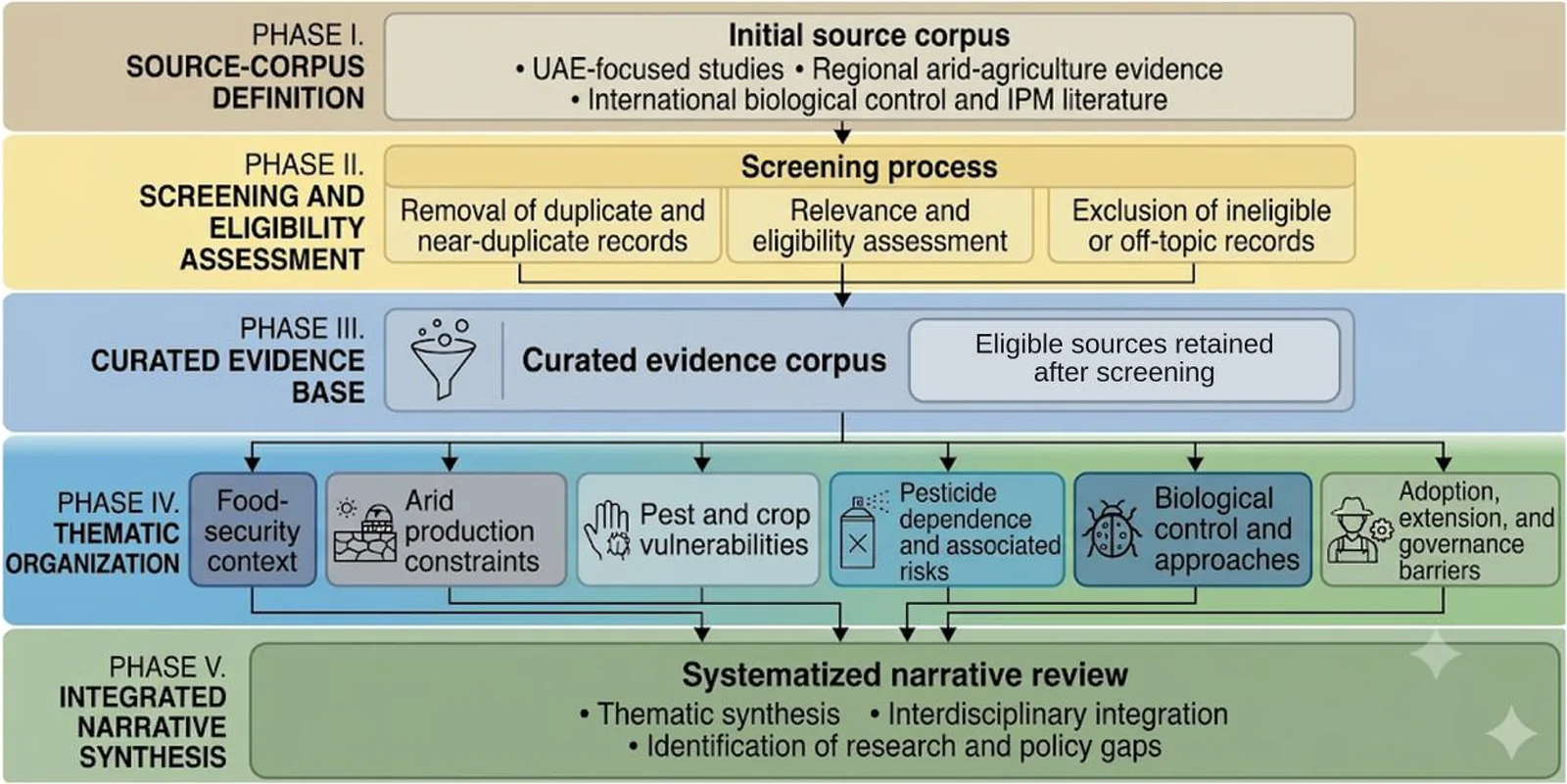
Review workflow and evidence-handling framework The workflow summarizes source-corpus definition, removal of duplicate and near-duplicate records, relevance and eligibility assessment, construction of the curated evidence corpus, thematic organization, and integrated narrative synthesis. Eligible sources were organized into six analytical domains: food-security context; arid production constraints; pest and crop vulnerabilities; pesticide dependence and associated risks; biological control and integrated pest management approaches; and adoption, extension, and governance barriers. See also Tables S1–S8.

The review workflow is summarized in Figure 1, and the main design features, eligibility criteria, and analytical procedures are summarized in Table 1. The contribution of the included sources to the principal themes of the review is documented in Tables S1–S8.

**Table 1:** Review design, evidence-selection criteria, and analytical strategy

Element	Description
Review design	Systematized narrative review designed to integrate heterogeneous evidence relevant to sustainable pest management in the UAE.
Evidence base	Curated corpus of peer-reviewed articles, reviews, modeling studies, field studies, and selected institutional and regional reports.
Geographic scope	UAE-specific evidence was prioritized. Gulf-region and international evidence was retained when directly relevant to arid UAE agricultural conditions.
Eligibility criteria	Sources were retained when they contributed to food security, arid production constraints, crop and pest vulnerability, pesticide-associated risks, biological control and IPM, or adoption and governance.
Exclusion criteria	Duplicate and near-duplicate records, clearly off-topic sources, and sources that did not materially inform the review question were excluded.
Screening procedure	Records underwent duplicate removal, relevance assessment against the review question and analytical domains, and thematic classification.
Data extraction	Information was extracted on study purpose, geographic focus, crop or production system, pest-management theme, main findings, and relevance to UAE sustainability transitions.
Evidence organization	Included sources were grouped into six thematic clusters: food-security context; arid production constraints; pest and crop vulnerabilities; pesticide dependence and associated risks; biological control and IPM approaches; and adoption, extension, and governance barriers.
Evidence appraisal	No numerical quality score was applied. Evidence was interpreted using a relevance-and-credibility framework that prioritized direct UAE relevance and clear links to sustainability outcomes.
Analytical approach	Thematic comparison and critical narrative synthesis were used instead of quantitative pooling or meta-analysis.
Methodological boundary	The review synthesizes a defined literature corpus and does not claim exhaustive coverage equivalent to a formal systematic review.

### Data extraction and evidence appraisal

Information was extracted from each included source on the study purpose, geographic focus, crop or production system, principal pest-management theme, main findings, and relevance to sustainable crop-protection transitions in the UAE. The extracted information was used to compare evidence across agronomy, entomology, plant pathology, environmental health, food safety, agricultural extension, and sustainability research.

A formal numerical risk-of-bias or quality-appraisal instrument was not applied because the evidence corpus contained substantially different study types. Instead, the review used a relevance-and-credibility framework. Greater interpretive weight was assigned to UAE-specific empirical studies, recent comprehensive reviews, and studies that explicitly connected pest-management practices with environmental quality, occupational health, food safety, biodiversity, soil function, water quality, or agricultural resilience. Older studies were retained when they provided uniquely relevant UAE evidence, foundational pest-management concepts, or information that had not been adequately addressed by more recent studies.

### Evidence synthesis

The extracted evidence was synthesized through thematic comparison and critical narrative integration. Cross-study comparison was used to identify recurrent drivers of pesticide dependence; major environmental, occupational, and food-safety concerns; feasible biological-control and IPM options under hot, saline, and water-limited conditions; and the technical, behavioral, institutional, regulatory, and policy barriers affecting adoption.

No pooled effect estimates were calculated because the included studies differed substantially in design, crop system, geographic setting, intervention, comparator, and reported outcome. Accordingly, this article should be interpreted as a transparent and structured synthesis of a defined evidence corpus rather than as an exhaustive systematic review or meta-analysis. The approach nevertheless provides a reproducible framework for integrating UAE-specific evidence with relevant regional and international scholarship and for identifying research priorities and practical pathways for sustainable pest management in UAE agriculture.

To support analytical coherence, the included studies were organized into six thematic clusters. The first cluster addressed the strategic importance of domestic agriculture for UAE food security. The second examined structural production constraints in arid agriculture, including water scarcity, salinity, land degradation, and climate stress. The third synthesized evidence on crop vulnerability and pest pressure, with emphasis on date palm, vegetable systems, and protected cultivation. The fourth focused on chemical pesticide dependence and its consequences, including resistance development, residue formation, ecological disruption, occupational exposure, and contamination risks. The fifth reviewed the technical scope of biological control and IPM, including microbial agents, semiochemicals, conservation biological control, monitoring approaches, and precision-support technologies. The sixth cluster examined farmers’ adoption, knowledge systems, extension capacity, institutional support, and policy conditions that shape the transition from chemical-intensive control toward more sustainable alternatives. These domains are consolidated in Table 1.

## Why agriculture still matters in the UAE

The importance of agriculture in the UAE is often understated when judged only by its share of gross domestic product (GDP) or total national employment. The literature instead frames agriculture as a strategic asset within a highly vulnerable food system. Sharma et al. (2024) position technology-driven domestic production as part of a broader effort to reduce import dependence and improve resilience to supply-chain disruption,^6^ while Ammar et al. (2024) argue that food security in the UAE must be understood in multidimensional terms, encompassing availability, access, utilization, and stability.^5^ From this perspective, agriculture matters not merely because of its economic contribution but also because it supports national resilience amid import exposure, environmental stress, and rising uncertainty. Pest management, therefore, should not be treated as a peripheral agronomic issue; it is directly linked to food-system security and strategic continuity in domestic production.

The source base also shows that UAE agriculture is highly differentiated. Date palm retains economic, ecological, and cultural significance, while vegetables, protected-horticulture systems, and selected cereal and tuber crops constitute additional production domains with distinct vulnerabilities. Manikandan et al. (2025) show that date palm cultivation in the Arabian Peninsula remains highly exposed to salinity, drought, temperature extremes, and pest pressure.^18^ Fatnassi et al. (2023) likewise demonstrate that protected agriculture offers important opportunities for food production in the UAE, although its performance remains constrained by extreme heat, water scarcity, and technological limitations.^21^ In addition, Alkaabi et al. (2025) show that farmers’ perceptions of land suitability and productivity are shaped by water management, crop choice, sustainability awareness, and knowledge-sharing networks. Taken together, these studies indicate that crop protection in the UAE must be understood within a broader sustainability transition rather than as an isolated technical problem.^20^

Beyond conventional crop production, the UAE also retains a significant ethnobotanical and medicinal-plant heritage that remains relevant to both local use and contemporary scientific research. Recent reviews document diverse medicinal plants native to, cultivated in, or widely used in the UAE, including *Lawsonia inermis*, *Nigella sativa*, *Ziziphus* spp., *Allium cepa*, *Allium sativum*, *Phoenix dactylifera*, *Trigonella foenum-graecum*, *Withania somnifera*, *Capparis spinosa*, *Citrullus colocynthis*, *Morus alba*, and *Rhazya stricta*. These plants are discussed in relation to antidiabetic, antimicrobial, cardioprotective, nutritional, and food-safety applications, showing that the agricultural value of UAE flora extends beyond staple and commercial crops to include medicinal species with continuing cultural and therapeutic relevance.^46^^,^^47^^,^^48^ This wider botanical base further strengthens the argument that agriculture in the UAE should be viewed not only as a production sector, but also as part of a broader health, sustainability, and bioresource system.^46^^,^^47^^,^^48^^,^^49^^,^^50^^,^^51^

## Pesticide-intensive crop protection and its sustainability limits

Chemical pesticides remain deeply embedded in modern agriculture because they are often effective, scalable, and immediately legible to farmers as a means of control. Reviews such as Tudi et al. (2021) and Shattuck et al. (2023) demonstrate how pesticide use has become structurally linked to the modernization of agriculture and global crop protection markets. However, these same reviews also show that the true environmental and social costs of pesticide-intensive systems are regularly externalized. When pest management is judged only by visible short-term suppression, it becomes easy to overlook the longer-term effects on biodiversity, ecological regulation, residues, resistance, and environmental contamination.^8^^,^^29^ The UAE-specific evidence, although still limited, points in the same direction. Kaakeh et al. (2007) documented extensive pesticide use in vegetable farms across the UAE and Oman, with farmers using large numbers of insecticides, fungicides, and a smaller range of herbicides. Particularly important is the finding that much pest-management information came from pesticide sales representatives. That pattern matters because it suggests that control decisions may be shaped less by ecological thresholds and more by product-centered advisory systems. In fragile arid agroecosystems, this can lock farms into input-intensive pest-management routines that are difficult to reverse.^16^

Pesticide dependence is also self-reinforcing. Pu and Chung (2024) show that insecticide resistance now involves multiple mechanisms, including target-site, metabolic, penetration, and sequestration-based processes.^52^ Sen et al. (2023) similarly describe herbicide resistance as a complex and evolving systems problem rather than a simple single-gene phenomenon.^53^ When such resistance dynamics are combined with the repeated intervention pressures typical of high-value crops, chemical dependency can produce a treadmill effect: pests become harder to control, treatment frequency rises, and selective pressure intensifies further. This is a particularly serious concern for the UAE, where heat-induced crop stress and water scarcity may already narrow the margin for agronomic error.

## Environmental, food-safety, and public-health consequences

A major finding across the literature is that pesticide impacts extend beyond target pests. Verdadero et al. (2025), Pathak et al. (2022), Zhou et al. (2025), and Kumar et al. (2024) describe effects on soil biota, non-target arthropods, aquatic systems, birds, and wider ecological interactions. These effects may be particularly consequential in arid agriculture, where ecological buffering capacity is limited. Reductions in beneficial organisms and biological regulation can, in turn, increase reliance on repeated chemical intervention.^28^^,^^54^^,^^55^^,^^56^

The public-health dimension is equally important. Shekhar et al. (2024) synthesize evidence linking chronic pesticide exposure to cancer, neurological disorders, respiratory problems, and endocrine disruption. Desye et al. (2024) show that unsafe use practices remain common among farmers in developing-country contexts, while Beyuo et al. (2024) and Jia et al. (2024) review the implications of residues in plant-derived and animal-derived foods.^42^^,^^57^^,^^58^ Shahidullah et al. (2023) further demonstrate that knowledge, attitude, and practice gaps can perpetuate unsafe pesticide use.^45^ While not all of these studies are UAE-specific, their relevance to the UAE is strong because the underlying causal pathways, including training deficits, exposure risk, and food-safety concerns, are highly transferable.

The environmental risks of pesticide-intensive agriculture also intersect with water and soil vulnerability. Chander et al. (2024) explain how intensive fertilizer and pesticide use can contribute to nitrate contamination in arid and semi-arid groundwater systems.^59^ Williams et al. (2025) show that land use and climate conditions increase groundwater vulnerability in arid contexts.^25^ Wang et al. (2023), P. Sharma et al. (2024), and Al-Shammary et al. (2024) collectively demonstrate that soil degradation, salinization, declining organic matter, and impaired microbial function are core risks in dryland agriculture. These findings are crucial for the UAE, where water quality, soil functionality, and long-term land productivity are already under structural pressure.^24^^,^^60^^,^^61^

## Pest pressures in UAE agriculture: Date palm, vegetables, and climate stress

The most prominent pest challenge in the UAE literature is the red palm weevil (Rhynchophorus ferrugineus), a major threat to date palm across the Gulf and wider palm-growing regions. Aziz (2024) reviews the global invasiveness and severity of the pest, emphasizing that control success remains uneven despite extensive management effort.^12^ In the UAE, Na et al. (2024) show that carefully selected combinations of pheromones, food bait, and ethyl acetate can substantially improve trapping performance.^19^ The same study also identifies a strong temporal pattern in adult activity, suggesting that better ecological understanding can improve intervention design. Hammami et al. (2024) report that organic treatment reduced weevil populations more effectively than chemical treatment in their long-running ICBA field context, while also highlighting the role of detection technologies, electronic monitoring, and future digital tools.^15^

Vegetable systems present a different but equally important challenge. Kaakeh et al. (2007) found that UAE farms faced a broad pest and disease complex, including whiteflies, leafminers, cutworms, tomato fruitworms, eggplant fruitworms, damping off, downy mildew, and blights. Such pest diversity increases uncertainty and can push growers toward prophylactic or frequent spraying.^16^ Yet that same diversity is precisely why IPM matters: diversified pest pressure is not managed well by simplistic, single-tactic pesticide strategies. Instead, it requires combinations of monitoring, threshold-based action, sanitation, selective chemistry, biological suppression, and crop-specific prevention measures.

Climate change and irrigation stress are likely to intensify both kinds of vulnerability. Mamassi et al. (2025) show that rising temperatures and water stress can sharply reduce crop yields and alter the resilience profiles of wheat, maize, and potato under future emissions scenarios in the UAE.^17^ Dzvene et al. (2025) further show, at the arid and semi-arid system level, that climate adaptation in agri-food systems will depend on strategic irrigation, crop diversification, and tolerant varieties.^62^ From a pest management perspective, this means future control strategies must be climate-aware. Heat stress can alter plant susceptibility, pest development rates, and biological control performance, making static pesticide calendars increasingly unfit for purpose.

## What biological control can realistically contribute

Biological control is sometimes presented too loosely in applied discussions, so conceptual precision is important. Stenberg et al. (2021) clarify that biological control involves living organisms that suppress pests either directly or indirectly, and that such control can occur through natural, conservation, classical, or augmentative pathways.^35^ This definition is useful because it distinguishes true biological control from broader low-chemical management rhetoric. For the UAE, biological control should therefore be understood as a family of living-agent strategies that can include microbial antagonists, parasitoids, predators, and ecological enhancement of resident natural enemies.

The literature demonstrates several mechanisms through which biological control may improve sustainability. Lahlali et al. (2022), Lee et al. (2023), Beyari (2025), Sarrocco (2023), Tyagi et al. (2024), and Nchu (2024) collectively describe direct antagonism, competition, induced systemic resistance, microbiome-mediated suppression, and disease regulation by beneficial organisms. These mechanisms are valuable in arid agriculture for at least three reasons. First, they reduce selective pressure for conventional pesticide resistance.^30^^,^^31^^,^^32^^,^^36^^,^^63^^,^^64^ Second, they can preserve beneficial biodiversity rather than eroding it. Third, they align well with residue-sensitive food systems and sustainability-oriented production narratives.

Still, biological control should not be romanticized. The reviewed literature is clear that efficacy can vary with temperature, humidity, crop system, ultraviolet exposure, application timing, and compatibility with other farm operations. Bai et al. (2023) remind us that biological control must be evaluated alongside physical and chemical control rather than assumed to dominate all contexts. In the UAE, where climatic harshness is a defining condition, that means locally validated deployment strategies are essential. The practical question is not whether biocontrol works somewhere, but under what conditions it works reliably in saline, hot, and water-limited systems.^65^

For date palm in particular, the outlook is promising but still developing. Recent Gulf-focused evidence indicates that microbial bioinsecticides, entomopathogenic nematodes, semiochemicals, smart monitoring tools, and other lower-impact approaches could improve insect management in date-palm systems under arid conditions.^22^ This suggests that red palm weevil management is one of the most realistic near-term entry points for scaling biocontrol-centered IPM in the UAE. The crop is strategic, the pest is severe, and the limitations of chemical-heavy approaches are already well recognized.

## Integrated pest management as the operative framework

The evidence reviewed here indicates that biological control is most effective when embedded within an IPM framework rather than treated as a stand-alone substitute for conventional pesticides. Deguine et al. (2021) show that many IPM programs have drifted away from their ecological foundations, leaving chemical control as the operational default.^38^ Tamò et al. (2022) address this limitation by proposing an “IPM 3.0” model centered on improved farmer decision-making, stronger nature-based options, and the integration of emerging technologies.^41^ This direction is reinforced by more recent synthesis work showing that contemporary IPM increasingly depends on biological control, conservation practices, targeted pesticide delivery, and stronger knowledge exchange among stakeholders.^9^^,^^37^^,^^66^ In that sense, the key issue for the UAE is not whether biological control can replace pesticides in every situation, but how biological, ecological, and chemical tactics can be combined in a more selective, knowledge-based, and sustainability-oriented crop-protection system.^39^^,^^40^

For the UAE, an operational IPM framework can be conceptualized around five interlinked elements. First, surveillance should rely on pheromone traps, field scouting, and digital monitoring to detect pest pressure early and improve the timing of intervention. Second, decision-making should be threshold-based, replacing routine spraying with responses linked to pest density, crop stage, and expected damage. Third, biological and ecological tactics should be expanded to include microbial products, predators, parasitoids, sanitation, habitat management, and attractant-based systems. Fourth, when chemical intervention is required, pesticide use should be precise, selective, and timed to minimize non-target damage and resistance pressure. Fifth, pest management must be integrated with water-smart and climate-smart agronomy so that stressed crops are not managed as though pest dynamics were independent of salinity, heat, or irrigation stress. This system’s logic is particularly relevant for UAE and Gulf date-palm and protected-cultivation systems, where sustainable insect management now increasingly combines biocontrol agents, semiochemicals, precision monitoring, and early-detection technologies.^12^^,^^14^^,^^15^^,^^20^^,^^21^^,^^22^^,^^26^^,^^67^

Precision agriculture can further strengthen this framework. Fleischer et al. (1999) established the logic of precision IPM as pest mapping for spatially variable management,^67^ and this approach has become increasingly relevant in arid agriculture, where remote sensing, Internet of Things systems, and smart irrigation can support site-specific decisions.^26^ In the UAE context, this is especially important because it offers a pathway away from blanket pesticide use and toward data-informed intervention that is both agronomically and environmentally defensible. Recent red palm weevil studies also support this direction by emphasizing electronic detection systems, semiochemical trapping, and integrated eco-friendly management packages for date palm under Gulf conditions.^15^^,^^22^ At the same time, implementation cannot be understood as a purely technical matter. Adoption studies show that knowledge, training, trust, incentives, and advisory systems remain.^42^^,^^44^^,^^68^ Accordingly, a credible UAE transition away from routine pesticide dependence will require not only ecological efficacy but also stronger extension, farmer training, and governance support.

Taken together, the literature suggests that the future of pest management in the UAE lies not in replacing one input with another, but in redesigning crop protection around IPM principles that combine surveillance, thresholds, biological control, selective pesticide use, and climate-smart agronomy. In this framework, biological control is not a peripheral add-on; it is a central component of a broader transition toward more resilient, knowledge-based, and resource-efficient agriculture.

## Adoption barriers, farmer behavior, and institutional constraints

A technically valid alternative does not automatically become an adopted one. One of the clearest themes in the literature reviewed is that sustainable pest management depends as much on institutions and behavior as on efficacy data. Russo et al. (2025) identify education, knowledge of specific practices, government support, and financial incentives as central drivers of adoption.^44^ Forouzani et al. (2024) show that perceived usefulness, ease of use, and farmer attitudes are powerful determinants of willingness to use biological inputs.^43^ The Saudi study on red palm weevil IPM adoption also indicates that even when IPM is promoted, uptake is uneven across control categories and farmer segments.^69^

These findings matter for the UAE because the local transition to biocontrol will almost certainly be constrained by similar adoption dynamics. Farmers and farm managers may perceive chemical pesticides as faster, simpler, or more reliable than biological tools, even when the long-term system costs favor the latter. Advisory structures also matter. If product distributors remain the main source of pest-management guidance, then market incentives may continue to support conventional inputs. By contrast, a transition toward biocontrol-centered IPM requires credible extension, demonstration effects, and context-specific evidence that alternative approaches can work under commercial conditions.

The KAP and extension literature reinforces this point. Studies from Bangladesh, Kenya, Uganda, Bhutan, Ethiopia, Saudi Arabia, and other smallholder settings consistently show that knowledge quality, label literacy, prior training, access to advisory services, and perceptions of risk shape whether growers continue with conventional pesticides or shift toward safer and more ecological options.^42^^,^^45^^,^^70^^,^^71^^,^^72^^,^^73^ The relevance for the UAE is not that these settings are identical, but that they highlight a common policy lesson: product substitution alone is insufficient without demonstration, extension, incentive alignment, and trusted information channels. There is also a regulatory dimension. Biocontrol agents, microbial products, and semiochemical systems often face different registration, validation, and commercialization pathways than conventional pesticides. Without predictable approval mechanisms, uptake remains fragmented. The literature on sustainable agriecosystems and digital agriecosystem orchestration further suggests that innovation diffusion depends on supporting services, not just products. In practice, this means that the UAE needs a support architecture around biocontrol, including testing, advisory translation, quality assurance, procurement signals, and residue-oriented market incentives.

## Toward a UAE-specific implementation framework

The evidence reviewed here suggests that the most appropriate transition pathway for the UAE is staged rather than abrupt. This conclusion follows from the convergence of several lines of evidence: the strong strategic value of date palm and protected systems, the ecological and residue-related limits of chemical dependence, the relevance of pheromone-based and early-detection approaches for red palm weevil, the need to adapt pest control to water scarcity and salinity, and the importance of farmer decision-making, training, and institutional support for successful implementation.^12^^,^^15^^,^^19^^,^^20^^,^^21^^,^^22^^,^^26^ This staged implementation logic is summarized in Table 2.

**Table 2:** Staged implementation roadmap for biocontrol-centered integrated pest management in the UAE

Time horizon	Operational focus	Illustrative actions
Near term	Improve detection and decision quality	Expand pheromone trapping, field scouting, early detection, extension campaigns, residue-awareness programs, and threshold-based spray decisions in date palm and vegetable systems.
Medium term	Scale validated alternatives	Register and promote microbial biocontrol agents, bioinsecticides, attract-and-kill systems, and protected-cultivation IPM packages adapted to hot and saline conditions.
Long term	Institutionalize ecological pest management	Link procurement, certification, and policy support to verified IPM performance; invest in precision monitoring, local biocontrol production, UAE-specific efficacy trials, and long-term extension support.

In practical terms, reform should begin in systems where the case for change is strongest, and implementation is most feasible, particularly date palm and protected horticulture. In the near term, priority should be given to improving detection and decision quality through field scouting, pheromone trapping, early warning, and better advisory support. In the medium term, validated biological-control products and ecological tactics should be scaled as part of complete crop-protection packages rather than promoted as isolated substitutes for synthetic pesticides. Over the longer term, sustainable pest management should be institutionalized through regulatory support, local efficacy testing, precision monitoring, and stronger links between verified IPM performance, product quality, and market incentives.^15^^,^^19^^,^^20^^,^^22^^,^^41^

This staged pathway is more realistic than a simplistic “replace chemicals with biology” narrative. Under UAE conditions, water scarcity, heat load, salinity, and the high value of vulnerable crops all increase the perceived cost of control failure. A credible policy pathway must therefore reduce transition risk for growers through demonstration farms, comparative efficacy trials, cost-effectiveness analysis, residue-linked quality incentives, and extension models that connect sustainability goals with profitability and product quality. In other words, ecological crop protection must be made operational rather than merely aspirational.

## Research gaps and future agenda

Several research gaps remain visible. First, the UAE still lacks a broad and recent empirical evidence base on farmer behavior, pest-management practice, and crop-specific pesticide decision-making. The Kaakeh et al. (2007) study remains valuable, but it cannot by itself describe the current policy and market environment.^16^ Updated surveys and mixed-method studies are needed. Second, the literature on biocontrol efficacy under UAE field conditions is still relatively narrow compared with the scale of the sustainability challenge. More work is needed on thermal tolerance, formulation stability, timing, compatibility with saline irrigation contexts, and integration with protected agriculture systems.

Third, adoption research should move beyond generic acceptance questions and toward practical transition design. Which incentives matter most for growers? How do farm size, market channel, and crop type affect willingness to adopt biological inputs? What role can certification, procurement standards, or digital advisory systems play? Fourth, there is a strong case for linking pest management to broader sustainability metrics, including water productivity, eco-efficiency, residue reduction, biodiversity retention, and soil function. Love et al. (2025) suggest one possible route through eco-efficiency thinking, although UAE-specific adaptation would be required.

Finally, the UAE would benefit from more interdisciplinary work that brings together entomology, plant pathology, soil science, irrigation management, behavioral science, and public policy. Biological control in arid agriculture is not only a pest problem.^40^ It is also a governance problem, a knowledge-transfer problem, and a systems-design problem. That is why the most useful future studies will likely be those that evaluate pest-management packages under realistic field conditions and connect agronomic performance to adoption feasibility and long-term sustainability outcomes.

## Conclusion

This review shows that the case for reducing pesticide dependence in UAE agriculture is both environmental and strategic. The country’s arid agroecosystems are too resource-constrained and too ecologically fragile to sustain an indefinitely chemical-intensive pest-management pathway without escalating costs. The literature links such dependence to resistance, non-target damage, residue concerns, occupational exposure, and threats to soil and water quality. These pressures are magnified by climate change and the strategic importance of domestic food production in an import-dependent nation.

Biological control offers a credible alternative, but only when understood in realistic terms. It is not a universal substitute for all pesticide use. Rather, it is a central component of a more sophisticated pest-management paradigm grounded in ecological regulation, better surveillance, targeted action, and compatibility with climate-smart farming. In the UAE, the most promising route forward is a biocontrol-centered IPM strategy, especially in date palm and protected-cultivation systems, supported by stronger extension, improved registration pathways, precision monitoring, and local validation of performance under hot and saline conditions.

The broader implication is that crop protection should be treated as part of food security and sustainability policy, not as a narrowly technical farm-input decision. If the UAE wishes to produce more food under arid conditions without deepening ecological stress, then pest management must evolve from routine chemical suppression to evidence-based ecological stewardship. Biological control is not the whole answer, but it is one of the most important pieces of the answer.

### Limitations of the study

This review has several limitations. First, the evidence corpus was assembled from a curated literature matrix rather than generated through a *de novo* search of multiple bibliographic databases; consequently, the review may not include every eligible publication. Second, the included evidence was heterogeneous in study design, geographic scope, crop system, intervention, and reported outcome, which precluded meta-analysis and limits direct comparison across studies. Third, a formal numerical risk-of-bias assessment was not applied, and the synthesis relied on relevance- and credibility-based interpretive weighting. Fourth, UAE-specific field evidence remains limited for several biological-control agents, crops, and implementation contexts, requiring cautious interpretation of evidence extrapolated from other arid and semi-arid regions. These limitations highlight the need for preregistered evidence syntheses, standardized outcome reporting, and additional UAE-based efficacy, adoption, and cost-effectiveness studies.

## Acknowledgments

The author received no specific funding for this work and has no additional acknowledgments.

## Author contributions

S.M.A.R.: conceptualization, methodology, investigation, data curation, formal analysis, visualization, writing – original draft, and writing – review and editing.

## Declaration of interests

The author declares no competing interests.

## Declaration of generative AI and AI-assisted technologies in the writing process

During the preparation and revision of this work, the author used ChatGPT (OpenAI) to assist with language editing and formatting. After using this tool, the author reviewed and edited the content as needed and takes full responsibility for the publication’s content.

## References

[1] Al Raish S.M., Abu-Elsaoud A.M., Askri A. (2026). Knowledge, attitudes, and practices of agrochemical use among farmers in the United Arab Emirates: a pre-and post-intervention assessment. Front. Sustain. Food Syst..

[2] Al Raish S.M., Abu-Elsaoud A.M., El-Tarabily K.A., AbuQamar S.F., Almasri R.S., Bedir A.S. (2026). Understanding drivers and barriers of organic vegetable consumption among adult residents in the United Arab Emirates: insights from knowledge, attitudes, and practices analysis. Front. Sustain. Food Syst..

[3] Al Raish S.M., Alsheriafi H.N., Alkuwaiti A.A., Safi S.K., Safi A.S. (2026). Insights into knowledge, attitudes, and practices of medicinal plant use in the United Arab Emirates: a cross-sectional study. Front. Nutr..

[4] Alteneiji S.M., Mathew B.T., Mohammed H.A., Abu-Elsaoud A.M., El-Tarabily K.A., Al Raish S.M. (2024). Knowledge, Attitudes, and Practices towards Single-Use Plastic Bags in the United Arab Emirates. Sustainability.

[5] Ammar K.A., Kheir A.M.S., Ali B.M., Sundarakani B., Manikas I. (2023). Developing an analytical framework for estimating food security indicators in the United Arab Emirates: A review. Environ. Dev. Sustain..

[6] Sharma R., Wahbeh S., Sundarakani B., Manikas I., Pachayappan M. (2024). Enhancing domestic food supply in the UAE: A framework for technology-driven urban farming systems. J. Clean. Prod..

[7] Shekhar C., Khosya R., Thakur K., Mahajan D., Kumar R., Kumar S., Sharma A.K. (2024). A systematic review of pesticide exposure, associated risks, and long-term human health impacts. Toxicol. Rep..

[8] Tudi M., Daniel Ruan H., Wang L., Lyu J., Sadler R., Connell D., Chu C., Phung D.T. (2021). Agriculture Development, Pesticide Application and Its Impact on the Environment. IJERPH.

[9] Zhou W., Arcot Y., Medina R.F., Bernal J., Cisneros-Zevallos L., Akbulut M.E.S. (2024). Integrated Pest Management: An Update on the Sustainability Approach to Crop Protection. ACS Omega.

[10] Al Raish S.M., Saeed E.E., Sham A., Alblooshi K., El-Tarabily K.A., AbuQamar S.F. (2020). Molecular Characterization and Disease Control of Stem Canker on Royal Poinciana (Delonix regia) Caused by Neoscytalidium dimidiatum in the United Arab Emirates. Int. J. Mol. Sci..

[11] Al Raish S.M., Saeed E.E., Alyafei D.M., El-Tarabily K.A., AbuQamar S.F. (2021). Evaluation of streptomycete actinobacterial isolates as biocontrol agents against royal poinciana stem canker disease caused by the fungal pathogen *Neoscytalidium dimidiatum*. Biol. Control.

[12] Aziz A.T. (2024). Red palm weevil, Rhynchophorus ferrugineus, a significant threat to date palm tree, global invasions, consequences, and management techniques. J. Plant Dis. Prot..

[13] El-Saadony M.T., Saad A.M., Soliman S.M., Salem H.M., Ahmed A.I., Mahmood M., El-Tahan A.M., Ebrahim A.A.M., Abd El-Mageed T.A., Negm S.H. (2022). Plant growth-promoting microorganisms as biocontrol agents of plant diseases: Mechanisms, challenges and future perspectives. Front. Plant Sci..

[14] Al Hamad B.M., Al Raish S.M., Ramadan G.A., Saeed E.E., Alameri S.S.A., Al Senaani S.S., AbuQamar S.F., El-Tarabily K.A. (2021). Effectiveness of Augmentative Biological Control of Streptomyces griseorubens UAE2 Depends on 1-Aminocyclopropane-1-Carboxylic Acid Deaminase Activity against Neoscytalidium dimidiatum. J. Fungi.

[15] Hammami Z., Krizhanovsky E., Elbattay A., Singh R.K. (2024). Study on the effectiveness of different control techniques for red palm weevil (*Rhynchophorous ferrugineus*). Khalifa International Award for Date Palm and Agricultural Innovation.

[16] Kaakeh W., Talukder F.A., Aldahmani J.H., Maraqa M., Deadman M.L., Al-Jabrid S.A., Al-Saadib A., Al-Raeesi A.A., Al Hasani H., Al-Subhi L. (2007). Assessment of pest and pesticide trends in vegetable crops in the United Arab Emirates and Sultanate of OMAN. Pak. J. Sci. Ind. Res..

[17] Mamassi A., Nhamo N., Gummadi S., Ammar K., Dhanhani M.A.H.A., Hashmi H.A.A., Bouras H., Accatino F., Zaaboul R. (2025). Climate change and irrigation management shape crop resilience in UAE arid agriculture: APSIM model-driven assessment. Agric. Water Manag..

[18] Manikandan S.K., Jenifer A D., Gowda N.K., Nair V., Al-Ruzouq R., Gibril M.B.A., Lamghari F., Klironomos J., Hmoudi M.A., Sheteiwy M., El-Keblawy A. (2025). Advancing date palm cultivation in the Arabian Peninsula and beyond: Addressing stress tolerance, genetic diversity, and sustainable practices. Agric. Water Manag..

[19] Na S.-M., Im G.-I., Lee W.-S., Kim D.-G. (2024). Assessment of Attractant Combinations for the Management of Red Palm Weevils (Rhynchophorus ferrugineus) in the United Arab Emirates. Insects.

[20] Alkaabi K., Anjum M.T., Hamed A., Younes O. (2025). Modeling farmers’ perception of agricultural practices and soil quality in arid regions of UAE: A PLS-SEM approach to sustainable land use. Environ. Chall..

[21] Fatnassi H., Zaaboul R., Elbattay A., Molina Aiz F.D., Valera D.L. (2023). Protected agriculture systems in the UAE: challenges and opportunities. In XXXI International Horticultural Congress.

[22] Lamine M., Khallef A., Zineb A.B., Daldoul S., Elleuch A., Mliki A., Sayadi S., Gargouri M. (2026). Sustainable insect management for date palm in arid Gulf agro-ecosystems: challenges, bioinsecticides, and emerging innovations. Int. J. Trop. Insect Sci..

[23] El-Rawy M., Batelaan O., Al-Arifi N., Alotaibi A., Abdalla F., Gabr M. (2023). Climate Change Impacts on Water Resources in Arid and Semi-Arid Regions: A Case Study in Saudi Arabia. Water.

[24] Wang K., Li J., Zhou Z., Zhang X.J. (2023). Editorial: Soil degradation and restoration in arid and semi-arid regions. Front. Environ. Sci..

[25] Williams S.A., Megdal S.B., Zuniga-Teran A.A., Quanrud D.M., Christopherson G. (2024). Mapping groundwater vulnerability in arid regions: A comparative risk assessment using modified DRASTIC models, land use, and climate change factors. Land.

[26] Xing Y., Wang X. (2024). Precision Agriculture and Water Conservation Strategies for Sustainable Crop Production in Arid Regions. Plants.

[27] Zhang Z., Zhu G., Peng X. (2024). Photorespiration in plant adaptation to environmental changes. Crop and Environment.

[28] Pathak V.M., Verma V.K., Rawat B.S., Kaur B., Babu N., Sharma A., Dewali S., Yadav M., Kumari R., Singh S. (2022). Current status of pesticide effects on environment, human health and it’s eco-friendly management as bioremediation: A comprehensive review. Front. Microbiol..

[29] Shattuck A., Werner M., Mempel F., Dunivin Z., Galt R. (2023). Global pesticide use and trade database (GloPUT): New estimates show pesticide use trends in low-income countries substantially underestimated. Glob. Environ. Change.

[30] Beyari E.A. (2024). Alternatives to chemical pesticides: the role of microbial biocontrol agents in phytopathogen management: a comprehensive review. J. Plant Pathol..

[31] Lahlali R., Ezrari S., Radouane N., Kenfaoui J., Esmaeel Q., El Hamss H., Belabess Z., Barka E.A. (2022). Biological Control of Plant Pathogens: A Global Perspective. Microorganisms.

[32] Lee J., Kim S., Jung H., Koo B.-K., Han J.A., Lee H.-S. (2023). Exploiting Bacterial Genera as Biocontrol Agents: Mechanisms, Interactions and Applications in Sustainable Agriculture. J. Plant Biol..

[33] Shahwar D., Mushtaq Z., Mushtaq H., Alqarawi A.A., Park Y., Alshahrani T.S., Faizan S. (2023). Role of microbial inoculants as bio fertilizers for improving crop productivity: A review. Heliyon.

[34] Sharma P., Gaur N., Kaushal M., Prasad R. (2021). Microbial Biotechnology in Crop Protection.

[35] Stenberg J.A., Sundh I., Becher P.G., Björkman C., Dubey M., Egan P.A., Friberg H., Gil J.F., Jensen D.F., Jonsson M. (2021). When is it biological control? A framework of definitions, mechanisms, and classifications. J. Pest. Sci..

[36] Tyagi A., Lama Tamang T., Kashtoh H., Mir R.A., Mir Z.A., Manzoor S., Manzar N., Gani G., Vishwakarma S.K., Almalki M.A., Ali S. (2024). A Review on Biocontrol Agents as Sustainable Approach for Crop Disease Management: Applications, Production, and Future Perspectives. Horticulturae.

[37] Angon P.B., Mondal S., Jahan I., Datto M., Antu U.B., Ayshi F.J., Islam M.S. (2023). Integrated Pest Management (IPM) in Agriculture and Its Role in Maintaining Ecological Balance and Biodiversity. Advances in Agriculture.

[38] Deguine J.-P., Aubertot J.-N., Flor R.J., Lescourret F., Wyckhuys K.A.G., Ratnadass A. (2021). Integrated pest management: good intentions, hard realities. Agron. Sustain. Dev..

[39] Lane D. (2025). Comparing Integrated Pest Management approaches: European Union vs. United States. J. Integr. Pest Manage..

[40] Love M., Magarey R.D., Holderman B.L., Carley D.S., Maggi F., Singer N. (2025). Tracking sustainability in crop pest management in the United States using an eco-efficiency index. Front. Insect Sci..

[41] Tamò M., Glitho I., Tepa-Yotto G., Muniappan R. (2022). How does IPM 3.0 look like (and why do we need it in Africa)?. Curr. Opin. Insect Sci..

[42] Desye B., Tesfaye A.H., Daba C., Alemseged E.A., Angaw Y., Ebrahim A.M., Natnael T., Hassen S., Woretaw L. (2024). Pesticide safe use practice and acute health symptoms, and associated factors among farmers in developing countries: a systematic review and meta-analysis of an epidemiological evidence. BMC Public Health.

[43] Forouzani M., Bondori A., Mombini A.S. (2024). Why are farmers reluctant to accept biological inputs? a structural equation model of technology adoption. Front. Sustain. Food Syst..

[44] Russo I., Vecchio R., Viscecchia R., Cembalo L., De Devitiis B. (2025). Factors affecting farmers’ adoption of sustainable pest management practices: A scoping review. Environ. Dev. Sustain..

[45] Shahidullah A.K.M., Islam A., Rahman M. (2023). Knowledge, attitude, and practice of pesticide use by vegetable growers in Bangladesh: a health literacy perspective in relation to non-communicable diseases. Front. Sustain. Food Syst..

[46] Almasri R., Bedir A., Al Raish S. (2025). Comprehensive Ethnopharmacological Analysis of Medicinal Plants in the UAE: Lawsonia inermis, Nigella sativa, Ziziphus spina-christi, Allium cepa, Allium sativum, Cymbopogon schoenanthus, Matricaria aurea, Phoenix dactylifera, Portulaca oleracea, Reichardia tingitana, Salvadora persica, Solanum lycopersicum, Trigonella foenum-graecum, Withania somnifera, and Ziziphus lotus. Nutrients.

[47] Bedir A.S., Almasri R.S., Al Raish S.M. (2025). Therapeutic efficacy of Nigella sativa and Ziziphus lotus: sustainable strategies for diabetes, antimicrobial resistance, and health treatment. Front. Nutr..

[48] Al Raish S.M., Almasri R.S., Bedir A.S. (2025). Ancient Remedies, Modern Medicine: A Review of Antidiabetic, Cardioprotective, and Antimicrobial Activities of Date Palm (Phoenix dactylifera), Tomato (Solanum lycopersicum), Fenugreek (Trigonella foenum-graecum), and Ashwagandha (Withania somnifera). Biology.

[49] Bedir A.S., Almasri R.S., Azar Y.O., Elnady R.E., Al Raish S.M. (2025). Exploring the Therapeutic Potential of Allium cepa and Allium sativum Extracts: Current Strategies, Emerging Applications, and Sustainability Utilization. Biology.

[50] Al Raish S.M. (2026). The Efficacy of Plant Extracts Against Key Food-Borne Pathogens: A Mechanistic, Applications, and Advances. Microorganisms.

[51] Al Raish S.M., Almasri R.S., Bedir A.S., Elkahwagy A.A. (2025). Phytochemical Composition, Bioactive Compounds, and Antidiabetic Potential of Four Medicinal Plants Native to the UAE: Capparis spinosa, Citrullus colocynthis, Morus alba, and Rhazya stricta. Biology.

[52] Pu J., Chung H. (2024). New and emerging mechanisms of insecticide resistance. Curr. Opin. Insect Sci..

[53] Sen M.K., Bhattacharya S., Bharati R., Hamouzová K., Soukup J. (2023). Comprehensive insights into herbicide resistance mechanisms in weeds: a synergistic integration of transcriptomic and metabolomic analyses. Front. Plant Sci..

[54] Verdadero F.X.D., Agarap A.Z., Macatingrao C.N.E., Ordonez I.A., Tavu L.E.J., Pires D., Balendres M.A.O. (2025). Pesticides in the Environment: Benefits, Harms, and Detection Methods. Science.

[55] Zhou W., Li M., Achal V. (2025). A comprehensive review on environmental and human health impacts of chemical pesticide usage. Emerging Contam..

[56] Kumar J.K.S., Monica S.S., Vinothkumar B., Suganthi A., Paramasivam M. (2021). Impact of pesticide exposure on environment and biodiversity: A review. Agric. Rev..

[57] Beyuo J., Sackey L.N.A., Yeboah C., Kayoung P.Y., Koudadje D. (2024). The implications of pesticide residue in food crops on human health: a critical review. Discov. Agric..

[58] Jia Q., Liao G.Q., Chen L., Qian Y.Z., Yan X., Qiu J. (2024). Pesticide residues in animal-derived food: Current state and perspectives. Food Chem..

[59] Chander S., Yadav S., Gupta A., Ali S., Negm A. (2024). Groundwater Quality and Geochemistry in Arid and Semi-Arid Regions.

[60] Al-Shammary A.A.G., Al-Shihmani L.S.S., Fernández-Gálvez J., Caballero-Calvo A. (2024). Optimizing sustainable agriculture: A comprehensive review of agronomic practices and their impacts on soil attributes. J. Environ. Manag..

[61] Sharma P., Sharma P., Thakur N. (2024). Sustainable farming practices and soil health: a pathway to achieving SDGs and future prospects. Discov. Sustain..

[62] Dzvene A.R., Zhou L., Slayi M., Dirwai T.L. (2025). A scoping review on challenges and measures for climate change in arid and semi-arid agri-food systems. Discov. Sustain..

[63] Sarrocco S. (2023). Biological Disease Control by Beneficial (Micro)Organisms: Selected Breakthroughs in the Past 50 Years. Phytopathology.

[64] Nchu F. (2024). Sustainable Biological Control of Pests: The Way Forward. Appl. Sci. (Basel)..

[65] Bai Y., Wang L., Yuan X. (2023). Pesticide control, physical control, or biological control? How to manage forest pests and diseases more effectively. Front. Ecol. Evol..

[66] Tiwari A.K. (2024). IPM Essentials: Combining Biology, Ecology, and Agriculture for Sustainable Pest Control. J. Adv. Biol. Biotechnol..

[67] Fleischer S.J., Blom P.E., Weisz R. (1999). Sampling in precision IPM: When the objective is a map. Phytopathology.

[68] Mweke A., Alexandersson E., Mulugeta T., Ilomo M., Kritzinger Q., Matsuanyane L., Onyango C.M. (2025). Knowledge, attitude, and practice (KAP) analysis of agricultural biologicals among smallholder farmers across three counties in Kenya. J. Agric. Food Res..

[69] Alotaibi B.A., Ahmed A., Al-Zaidi A.A., Kassem H.S. (2022). Adoption of Integrated Pest Management for Red Palm Weevil Control among Farmers in Saudi Arabia. Horticulturae.

[70] Al-Sarar A.S., Bayoumi A.E., Herab A.H., Alzabib A.A., Bazeyad A.Y., Abobakr Y. (2025). Knowledge and attitude of vegetable farmers toward pesticide labels in Saudi Arabia: a cross-sectional study. Int. J. Agric. Sustain..

[71] Coria J., Elgueta S. (2022). Towards safer use of pesticides in Chile. Environ. Sci. Pollut. Res..

[72] Prajapati C.S., Priya N.K., Bishnoi S., Vishwakarma S.K., Buvaneswari K., Shastri S., Tripathi S., Jadhav A. (2025). The Role of Participatory Approaches in Modern Agricultural Extension: Bridging Knowledge Gaps for Sustainable Farming Practices. J. Exp. Agric. Int..

[73] Tonnang H.E.Z., Tepa-Yotto G.T., Sokame B.M., Winsou J.K., Tamò M., Djouaka R.F. (2025). Roadmap for mainstreaming integrated pest management (IPM) into a climate smart one-health (CS-OH) framework. One Health.

