# Supplementary material for: From chemical control to ecological resilience: Biological control and integrated pest management in arid agriculture
Source: iScience. 2026 Sep 1;29(9):117334. doi: 10.1016/j.isci.2026.117334 (PMC13562422; doi:10.1016/j.isci.2026.117334)
Supplement: Supplementary file 1 [file mmc1.pdf]

iScience, Volume *29*

Supplemental information

From chemical control to ecological resilience: Biological control and integrated pest management in arid agriculture

Seham M. Al Raish

Supplemental information.

Tables S1–S8 summarize the thematic contribution of sources included in the curated evidence corpus. Studies are grouped according to their principal analytical role; some sources contribute to more than one theme.

Table S1. Studies informing the UAE and GCC agri-food context, related to Section 3

Reference	Study title	Role in synthesis
**[S1]**	Enhancing domestic food supply in the UAE: A framework for technology-driven urban farming systems	Provides UAE/GCC agri-food systems context.
**[S2]**	Developing an analytical framework for estimating food security indicators in the United Arab Emirates: A review	Provides UAE/GCC agri-food systems context.
**[S3]**	Climate change and irrigation management shape crop resilience in UAE arid agriculture: APSIM model-driven assessment	Provides UAE/GCC agri-food systems context.
**[S4]**	Protected agriculture systems in the UAE: challenges and opportunities	Provides UAE/GCC agri-food systems context.
**[S5]**	Conceptualization of arid region radioecology strategies for agricultural ecosystems of the United Arab Emirates (UAE)	Provides UAE/GCC agri-food systems context.
**[S6]**	Greening the Desert: Sustainability Challenges and Environmental Initiatives in the GCC States	Supports the conceptual framing of biological disease control discussed in Section 7.
**[S7]**	Sustainability disclosure in the Gulf Cooperation Council (GCC) countries: Opportunities and Challenges	Provides broader GCC sustainability-governance context relevant to regional transition framing.

Table S2. Studies informing UAE and GCC pest management, related to Sections 6–10

Reference	Study title	Role in synthesis
**[S8]**	Advancing date palm cultivation in the Arabian Peninsula and beyond: Addressing stress tolerance, genetic diversity, and sustainable practices	Provides direct evidence on pest pressure or regional management responses.
**[S9]**	Evaluation of date palm fruits quality under different irrigation water salinity levels compared to the fruit available in the market	Provides direct evidence on date-palm system vulnerability.
**[S10]**	Assessment of Attractant Combinations for the Management of Red Palm Weevils (*Rhynchophorus ferrugineus*) in the United Arab Emirates	Provides direct evidence on regional pest-management responses.
**[S11]**	Study on the effectiveness of different control techniques for red palm weevil (*Rhynchophorous ferrugineus*)	Provides direct evidence on regional pest-management responses.
**[S12]**	Assessment of pest and pesticide trends in vegetable crops in the United Arab Emirates and Sultanate of Oman	Provides direct evidence on pest pressure and pesticide use in UAE vegetable systems.

**[S13]**	Red palm weevil, *Rhynchophorus ferrugineus*, a significant threat to date palm tree, global invasions, consequences, and management techniques	Provides direct evidence on date-palm pest pressure and management responses.
**[S14]**	Adoption of Integrated Pest Management for Red Palm Weevil Control among Farmers in Saudi Arabia	Provides Gulf-region implementation evidence relevant to date-palm pest management.
**[S15]**	In Vitro Detection of Acaricide Resistance in *Hyalomma* Species Ticks with Emphasis on Farm Management Practices Associated with Acaricide Resistance in Abu Dhabi, United Arab Emirates	Provides direct evidence on resistance and farm-management challenges in the UAE.
**[S16]**	Sustainable insect management for date palm in arid Gulf agro-ecosystems: Challenges, bioinsecticides, and emerging innovations	Provides direct evidence on Gulf-region sustainable pest-management responses.
**[S17]**	Effectiveness of Augmentative Biological Control of *Streptomyces griseorubens* UAE2 Depends on 1-Aminocyclopropane-1-Carboxylic Acid Deaminase Activity against *Neoscytalidium dimidiatum*	Provides UAE evidence on microbial biological control of a fungal stem-canker disease in royal poinciana.

Table S3. Studies informing arid-environment constraints, related to Sections 3, 5, 6, and 10

Reference	Study title	Role in synthesis
**[S18]**	Modeling farmers' perception of agricultural practices and soil quality in arid regions of UAE: A PLS-SEM approach to sustainable land use	Explains arid-environment constraints relevant to pest-management feasibility.
**[S19]**	Evaluation of Different Modelling Techniques with Fusion of Satellite, Soil and Agro-Meteorological Data for the Assessment of Durum Wheat Yield under a Large Scale Application	Explains arid-environment constraints relevant to pest-management feasibility.
**[S20]**	Investigating agricultural water sustainability in arid regions with Bayesian network and water footprint theories	Explains arid-environment constraints relevant to pest-management feasibility.
**[S21]**	Precision Agriculture and Water Conservation Strategies for Sustainable Crop Production in Arid Regions	Explains arid-environment constraints relevant to pest-management feasibility.
**[S22]**	A scoping review on challenges and measures for climate change in arid and semi-arid agri-food systems	Explains arid-environment constraints relevant to pest-management feasibility.
**[S23]**	Climate Change Impacts on Water Resources in Arid and Semi-Arid Regions: A Case Study in Saudi Arabia	Explains arid-environment constraints relevant to pest-management feasibility.
**[S24]**	Sustainable farming practices and soil health: A pathway to achieving SDGs and future prospects	Explains arid-environment constraints relevant to pest-management feasibility.
**[S25]**	Optimizing sustainable agriculture: A comprehensive review of agronomic practices and their impacts on soil attributes	Explains arid-environment constraints relevant to pest-management feasibility.
**[S26]**	Editorial: Soil degradation and restoration in arid and semi-arid regions	Explains arid-environment constraints relevant to pest-management feasibility.

**[S27]**	Environmental pollution and associated ecological risk assessment of the agricultural water in the arid coastal ecosystem	Explains arid-environment constraints relevant to pest-management feasibility.
**[S28]**	Nitrate Contamination in Groundwater of Arid and Semi-Arid Regions, Ecotoxicological Impacts, and Management Strategies	Explains arid-environment constraints relevant to pest-management feasibility.
**[S29]**	Mapping Groundwater Vulnerability in Arid Regions: A Comparative Risk Assessment Using Modified DRASTIC Models, Land Use, and Climate Change Factors	Explains arid-environment constraints relevant to pest-management feasibility.

Table S4. Studies informing pesticide impacts, safety, and resistance, related to Sections 4 and 5

Reference	Study title	Role in synthesis
**[S30]**	FAOSTAT pesticides use and trade highlights, 1990–2023	Documents pesticide reliance, residues, resistance, or ecological and health risks.
**[S31]**	Global pesticide use and trade database (GloPUT): New estimates show pesticide use trends in low-income countries substantially underestimated	Documents pesticide reliance, residues, resistance, or ecological and health risks.
**[S32]**	A comprehensive review on environmental and human health impacts of chemical pesticide usage	Documents pesticide reliance, residues, resistance, or ecological and health risks.
**[S33]**	Agriculture Development, Pesticide Application and Its Impact on the Environment	Documents pesticide reliance, residues, resistance, or ecological and health risks.
**[S34]**	Pesticides in the Environment: Benefits, Harms, and Detection Methods	Documents pesticide reliance, residues, resistance, or ecological and health risks.
**[S35]**	Impact of Pesticide Exposure on Environment and Biodiversity: A Review	Documents pesticide reliance, residues, resistance, or ecological and health risks.
**[S36]**	New and emerging mechanisms of insecticide resistance	Documents pesticide resistance dynamics that motivate transition.
**[S37]**	Comprehensive insights into herbicide resistance mechanisms in weeds: A synergistic integration of transcriptomic and metabolomic analyses	Documents pesticide resistance dynamics that motivate transition.
**[S38]**	Insecticide Resistance: Challenges and Solutions for Sustainable Pest Management	Documents pesticide resistance dynamics that motivate transition.
**[S39]**	A systematic review of pesticide exposure, associated risks, and long-term human health impacts	Documents human-health risks associated with pesticide dependence.
**[S40]**	Pesticide safe use practice and acute health symptoms, and associated factors among farmers in developing countries: A systematic review and meta-analysis of epidemiological evidence	Documents human-health risks and safe-use gaps associated with pesticide dependence.

**[S41]**	The implications of pesticide residue in food crops on human health: A critical review	Documents food-safety and residue concerns that motivate transition.
**[S42]**	Pesticide residues in animal-derived food: Current state and perspectives	Documents food-safety and residue concerns that motivate transition.
**[S43]**	Current status of pesticide effects on environment, human health and its eco-friendly management as bioremediation: A comprehensive review	Documents ecological and health risks while linking them to sustainable alternatives.

Table S5. Studies informing biological control and biological inputs, related to Section 7

Reference	Study title	Role in synthesis
**[S44]**	When is it biological control? A framework of definitions, mechanisms, and classifications	Explains mechanisms, benefits, or constraints of biological control and biological inputs.
**[S45]**	A Review on Biocontrol Agents as Sustainable Approach for Crop Disease Management: Applications, Production, and Future Perspectives	Explains mechanisms, benefits, or constraints of biological control and biological inputs.
**[S46]**	Sustainable Biological Control of Pests: The Way Forward	Explains mechanisms, benefits, or constraints of biological control and biological inputs.
**[S47]**	Exploiting Bacterial Genera as Biocontrol Agents: Mechanisms, Interactions and Applications in Sustainable Agriculture	Explains mechanisms, benefits, or constraints of biological control and biological inputs.
**[S48]**	Biological Control of Plant Pathogens: A Global Perspective	Explains mechanisms, benefits, or constraints of biological control and biological inputs.
**[S49]**	Alternatives to chemical pesticides: The role of microbial biocontrol agents in phytopathogen management: A comprehensive review	Explains mechanisms, benefits, or constraints of biological control and biological inputs.
**[S50]**	Microbial Biopesticides Use in Insect-Pest Management: An Overview	Explains mechanisms, benefits, or constraints of biological control and biological inputs.
**[S51]**	Role of microbial inoculants as biofertilizers for improving crop productivity: A review	Supports the broader biological-input perspective relevant to sustainable crop protection.
**[S52]**	Benefits associated with the implementation of biological control programmes in Latin America	Supports comparative interpretation of biological-control implementation.

Table S6. Studies informing integrated pest management, related to Section 8

Reference	Study title	Role in synthesis
**[S53]**	Sampling in Precision IPM: When the Objective Is a Map	Provides operational IPM concepts, architecture, or monitoring logic relevant to transition.
**[S54]**	Integrated pest management: Good intentions, hard realities. A review	Provides operational IPM concepts and critical evaluation of implementation.
**[S55]**	Integrated Pest Management (IPM) in Agriculture and Its Role in Maintaining Ecological Balance and Biodiversity	Provides operational IPM concepts and sustainability framing.
**[S56]**	How does IPM 3.0 look like (and why do we need it in Africa)?	Provides operational IPM concepts and modernization logic relevant to transition.
**[S57]**	Comparing Integrated Pest Management approaches: European Union vs. United States	Provides comparative IPM framework evidence relevant to transition.
**[S58]**	Integrated Pest Management: An Update on the Sustainability Approach to Crop Protection	Provides operational IPM concepts and contemporary sustainability framing.
**[S59]**	Tracking sustainability in crop pest management in the United States using an eco-efficiency index	Provides comparative IPM sustainability metrics relevant to transition.
**[S60]**	Advancing integrated pest management: Strategies for minimizing pesticide use	Provides operational IPM concepts relevant to pesticide-reduction pathways.

Table S7. Studies informing adoption, behavior, and extension, related to Section 9

Reference	Study title	Role in synthesis
**[S61]**	How to Successfully Orchestrate Content for Digital Agriecosystems	Helps interpret digital support systems and implementation pathways.
**[S62]**	Factors affecting farmers' adoption of sustainable pest management practices: A scoping review	Helps interpret adoption barriers and implementation pathways.
**[S63]**	Smallholder farmers' knowledge, attitudes, and practices (KAP) regarding agricultural inputs with a focus on agricultural biologicals	Helps interpret farmer perceptions and KAP evidence relevant to implementation.
**[S64]**	Knowledge, attitude, and practice (KAP) analysis of agricultural biologicals among smallholder farmers across three counties in Kenya	Helps interpret farmer perceptions and KAP evidence relevant to implementation.
**[S65]**	Why are farmers reluctant to accept biological inputs? A structural equation model of technology adoption	Helps interpret adoption barriers to biological inputs.
**[S66]**	Farmer perceptions and their implications for adoption of sustainable agricultural intensification practices	Helps interpret farmer perceptions and implementation barriers.
**[S40]**	Pesticide safe use practice and acute health symptoms, and associated factors among farmers in developing countries: A systematic review and meta-analysis of epidemiological evidence	Helps interpret knowledge, training, and implementation barriers linked to pesticide transition.

**[S67]**	Knowledge, attitude, and practice of pesticide use by vegetable growers in Bangladesh: A health literacy perspective in relation to non-communicable diseases	Helps interpret KAP evidence relevant to pesticide-use behavior.

Table S8. Additional supporting literature, related to Sections 3, 7, and 9

Reference	Study title	Role in synthesis
**[S68]**	Biological Disease Control by Beneficial (Micro)Organisms: Selected Breakthroughs in the Past 50 Years	Supports conceptual framing or comparative interpretation.
**[S7]**	Sustainability disclosure in the Gulf Cooperation Council (GCC) countries: Opportunities and Challenges	Supports broader GCC sustainability-governance framing.
**[S69]**	Sustainable development in the Arab world – is the Gulf Cooperation Council (GCC) region fit for the challenge?	Supports broader GCC sustainability framing and regional comparative interpretation.

Supplemental references

[S1] Sharma, R., Wahbeh, S., Sundarakani, B., Manikas, I., and Pachayappan, M. (2024). Enhancing domestic food supply in the UAE: A framework for technology-driven urban farming systems. Journal of Cleaner Production 434, 139823. doi: 10.1016/j.jclepro.2023.139823.

[S2] Ammar, K.A., Kheir, A.M.S., Ali, B.M., Sundarakani, B., and Manikas, I. (2024). Developing an analytical framework for estimating food security indicators in the United Arab Emirates: A review. Environ. Dev. Sustain. 26, 5689–5708. doi: 10.1007/s10668-023-03032-3.

[S3] Mamassi, A., Nhamo, N., Gummadi, S., Ammar, K., Dhanhani, M.A.H.A., Hashmi, H.A.A., Bouras, H., Accatino, F., and Zaaboul, R. (2025). Climate change and irrigation management shape crop resilience in UAE arid agriculture: APSIM model-driven assessment. Agricultural Water Management 319, 109819. doi: 10.1016/j.agwat.2025.109819.

[S4] Fatnassi, H., Zaaboul, R., Elbattay, A., Molina Aiz, F.D., and Valera, D.L. (2023). Protected agriculture systems in the UAE: Challenges and opportunities. International Center for Biosaline Agriculture.

[S5] Raj, P., Padiyath, N., Semioshkina, N., Addad, Y., Foulon, F., Francis, D., and Voigt, G. (2022). Conceptualization of arid region radioecology strategies for agricultural ecosystems of the United Arab Emirates (UAE). Science of the Total Environment 832, 154965. doi: 10.1016/j.scitotenv.2022.154965.

[S6] Alam, M., and Azalie, I.A.N. (2023). Greening the desert: Sustainability challenges and environmental initiatives in the GCC states. In Social Change in the Gulf Region: Multidisciplinary Perspectives, M.M. Rahman and A. Al-Azm, eds. (Springer Nature), pp. 493–510. doi: 10.1007/978-981-19-7796-1_29.

[S7] ElAlfy, A., Elgharbawy, A., Driver, T.R., and Ibrahim, A.-J. (2025). Sustainability disclosure in the Gulf Cooperation Council (GCC) countries: Opportunities and Challenges. Green Finance 7, 40–82. doi: 10.3934/GF.2025003.

[S8] Manikandan, S.K., Jenifer. A, D., Gowda, N.K., Nair, V., Al-Ruzouq, R., Gibril, M.B.A., Lamghari, F., Klironomos, J., Hmoudi, M.A., Sheteiwy, M., et al. (2025). Advancing date palm cultivation in the Arabian Peninsula and beyond: Addressing stress tolerance, genetic diversity, and sustainable practices. Agricultural Water Management 307, 109242. doi: 10.1016/j.agwat.2024.109242.

[S9] Hammami, Z., Mahmoudi, H., Al Janaahi, A., and Singh, R.K. (2024). Evaluation of date palm fruits quality under different irrigation water salinity levels compared to the fruit available in the market. Front. Sustain. Food Syst. 7, 1322350. doi: 10.3389/fsufs.2023.1322350.

[S10] Na, S.-M., Im, G.-I., Lee, W.-S., and Kim, D.-G. (2024). Assessment of attractant combinations for the management of red palm weevils (Rhynchophorus ferrugineus) in the United Arab Emirates. Insects 15, 218. doi: 10.3390/insects15040218.

[S11] Hammami, Z., Krizhanovsky, E., Elbattay, A., and Singh, R.K. (2024). Study on the effectiveness of different control techniques for red palm weevil (Rhynchophorous ferrugineus). Khalifa International Award for Date Palm and Agricultural Innovation 16, 48–61. https://www.biosaline.org/publications/study-effectiveness-different-control-techniques-red-palm-weevil-rhynchophorous.

[S12] Kaakeh, W., Talukder, F.A., Aldahmani, J.H., Maraqa, M., Deadman, M.L., Al-Jabrid, S.A., Al-Saadib, A., Al-Raeesi, A.A., Al Hasani, H., Al-Subhi, L., et al. (2007). Assessment of pest and pesticide trends in vegetable crops in the United Arab Emirates and Sultanate of Oman. Pak. J. Sci. Ind. Res. 50, 346–351.

[S13] Aziz, A.T. (2024). Red palm weevil, Rhynchophorus ferrugineus, a significant threat to date palm tree, global invasions, consequences, and management techniques. J. Plant Dis. Prot. 131, 9–26. doi: 10.1007/s41348-023-00805-w.

[S14] Alotaibi, B.A., Ahmed, A., Al-Zaidi, A.A., and Kassem, H.S. (2022). Adoption of integrated pest management for red palm weevil control among farmers in Saudi Arabia. Horticulturae 8, 1005. doi: 10.3390/horticulturae8111005.

[S15] Habeeba, S., Mahmmod, Y., Mohammed, H., Amer, H., Moustafa, M., Sobhi, A., El-Sokary, M., Hussein, M., Tolba, A., Al Hammadi, Z., et al. (2025). In vitro detection of acaricide resistance in Hyalomma species ticks with emphasis on farm management practices associated with acaricide resistance in Abu Dhabi, United Arab Emirates. Vet. Sci. 12, 712. doi: 10.3390/vetsci12080712.

[S16] Lamine, M., Khallef, A., Zineb, A.B., Daldoul, S., Elleuch, A., Mliki, A., Sayadi, S., and Gargouri, M. (2026). Sustainable insect management for date palm in arid Gulf agro-ecosystems: Challenges, bioinsecticides, and emerging innovations. Int. J. Trop. Insect Sci. 46, 885–899. doi: 10.1007/s42690-026-01783-w.

[S17] Al Hamad, B.M., Al Raish, S.M., Ramadan, G.A., Saeed, E.E., Alameri, S.S.A., Al Senaani, S.S., AbuQamar, S.F., and El-Tarabily, K.A. (2021). Effectiveness of augmentative biological control of Streptomyces griseorubens UAE2 depends on 1-aminocyclopropane-1-carboxylic acid deaminase activity against Neoscytalidium dimidiatum. J. Fungi 7, 885. doi: 10.3390/jof7110885.

[S18] Alkaabi, K., Anjum, M.T., Hamed, A., and Younes, O. (2025). Modeling farmers' perception of agricultural practices and soil quality in arid regions of UAE: A PLS-SEM approach to sustainable land use. Environmental Challenges 20, 101283. doi: 10.1016/j.envc.2025.101283.

[S19] Lekakis, E., Zaikos, A., Polychronidis, A., Efthimiou, C., Pourikas, I., and Mamouka, T. (2022). Evaluation of different modelling techniques with fusion of satellite, soil and agro-meteorological data for the assessment of durum wheat yield under a large-scale application. Agriculture 12, 1635. doi: 10.3390/agriculture12101635.

[S20] Zhang, L., Yu, Y., Guo, Z., Ding, X., Zhang, J., and Yu, R. (2024). Investigating agricultural water sustainability in arid regions with Bayesian network and water footprint theories. Sci. Total Environ. 951, 175544. doi: 10.1016/j.scitotenv.2024.175544.

[S21] Xing, Y., and Wang, X. (2024). Precision agriculture and water conservation strategies for sustainable crop production in arid regions. Plants 13, 3184. doi: 10.3390/plants13223184.

[S22] Dzvene, A.R., Zhou, L., Slayi, M., and Dirwai, T.L. (2025). A scoping review on challenges and measures for climate change in arid and semi-arid agri-food systems. Discov. Sustain. 6, 151. doi: 10.1007/s43621-025-00945-z.

[S23] El-Rawy, M., Batelaan, O., Al-Arifi, N., Alotaibi, A., Abdalla, F., and Gabr, M.E. (2023). Climate change impacts on water resources in arid and semi-arid regions: A case study in Saudi Arabia. Water 15, 606. doi: 10.3390/w15030606.

[S24] Sharma, P., Sharma, P., and Thakur, N. (2024). Sustainable farming practices and soil health: A pathway to achieving SDGs and future prospects. Discov. Sustain. 5, 250. doi: 10.1007/s43621-024-00447-4.

[S25] Al-Shammary, A.A.G., Al-Shihmani, L.S.S., Fernández-Gálvez, J., and Caballero-Calvo, A. (2024). Optimizing sustainable agriculture: A comprehensive review of agronomic practices and their impacts on soil attributes. J. Environ. Manage. 364, 121487. doi: 10.1016/j.jenvman.2024.121487.

[S26] Wang, K., Li, J., Zhou, Z., and Zhang, X.J. (2023). Editorial: Soil degradation and restoration in arid and semi-arid regions. Front. Environ. Sci. 11, 1307500. doi: 10.3389/fenvs.2023.1307500.

[S27] Benaafi, M., Tawabini, B., Salhi, B., Baalousha, H.M., Rahman, M., Hussain, I., and Aljundi, I.H. (2026). Environmental pollution and associated ecological risk assessment of the agricultural water in the arid coastal ecosystem. J. King Saud Univ. Sci. 38, 10382025. doi: 10.25259/JKSUS_1038_2025.

[S28] Chander, S., Yadav, S., and Gupta, A. (2024). Nitrate contamination in groundwater of arid and semi-arid regions, ecotoxicological impacts, and management strategies. In Groundwater Quality and Geochemistry in Arid and Semi-Arid Regions, S. Ali and A. Negm, eds. (Springer Nature Switzerland), pp. 339–369. doi: 10.1007/698_2023_1047.

[S29] Williams, S.A., Megdal, S.B., Zuniga-Teran, A.A., Quanrud, D.M., and Christopherson, G. (2025). Mapping groundwater vulnerability in arid regions: A comparative risk assessment using modified DRASTIC models, land use, and climate change factors. Land 14, 58. doi: 10.3390/land14010058.

[S30] FAO (2025). Pesticides use and trade, 1990–2023. FAOSTAT Analytical Brief No. 109 (Food and Agriculture Organization of the United Nations). https://openknowledge.fao.org/handle/20.500.14283/cd5968en.

[S31] Shattuck, A., Werner, M., Mempel, F., Dunivin, Z., and Galt, R. (2023). Global pesticide use and trade database (GloPUT): New estimates show pesticide use trends in low-income countries substantially underestimated. Glob. Environ. Change 81, 102693. doi: 10.1016/j.gloenvcha.2023.102693.

[S32] Zhou, W., Li, M., and Achal, V. (2025). A comprehensive review on environmental and human health impacts of chemical pesticide usage. Emerg. Contam. 11, 100410. doi: 10.1016/j.emcon.2024.100410.

[S33] Tudi, M., Daniel Ruan, H., Wang, L., Lyu, J., Sadler, R., Connell, D., Chu, C., and Phung, D.T. (2021). Agriculture development, pesticide application and its impact on the environment. Int. J. Environ. Res. Public Health 18, 1112. doi: 10.3390/ijerph18031112.

[S34] Verdadero, F.X.D., Agarap, A.Z., Macatingrao, C.N.E., Ordonez, I.A., Tavu, L.E.J., Pires, D., and Balendres, M.A.O. (2025). Pesticides in the environment: Benefits, harms, and detection methods. Sci 7, 171. doi: 10.3390/sci7040171.

[S35] Kumar, J.K.S., Monica, S.S., Vinothkumar, B., Suganthi, A., and Paramasivam, M. (2024). Impact of pesticide exposure on environment and biodiversity: A review. Agric. Rev. 45, 1–12. doi: 10.18805/ag.R-2325.

[S36] Pu, J., and Chung, H. (2024). New and emerging mechanisms of insecticide resistance. Curr. Opin. Insect Sci. 63, 101184. doi: 10.1016/j.cois.2024.101184.

[S37] Sen, M.K., Bhattacharya, S., Bharati, R., Hamouzová, K., and Soukup, J. (2023). Comprehensive insights into herbicide resistance mechanisms in weeds: A synergistic integration of transcriptomic and metabolomic analyses. Front. Plant Sci. 14, 1280118. doi: 10.3389/fpls.2023.1280118.

[S38] Tamta, A.K., and Srivastava, R.P. (2026). Insecticide resistance: Challenges and solutions for sustainable pest management. In Advancements in Entomology, D. Mitra, M. Pellegrini, and B.E.G. Sierra, eds. (Springer Nature Singapore), pp. 299–344. doi: 10.1007/978-981-95-5214-6_14.

[S39] Shekhar, C., Khosya, R., Thakur, K., Mahajan, D., Kumar, R., Kumar, S., and Sharma, A.K. (2024). A systematic review of pesticide exposure, associated risks, and long-term human health impacts. Toxicol. Rep. 13, 101840. doi: 10.1016/j.toxrep.2024.101840.

[S40] Desye, B., Tesfaye, A.H., Daba, C., Alemseged, E.A., Angaw, Y., Ebrahim, A.M., Natnael, T., Hassen, S., and Woretaw, L. (2024). Pesticide safe use practice and acute health symptoms, and associated factors among farmers in developing countries: A systematic review and meta-analysis of an epidemiological evidence. BMC Public Health 24, 3313. doi: 10.1186/s12889-024-20817-x.

[S41] Beyuo, J., Sackey, L.N.A., Yeboah, C., Kayoung, P.Y., and Koudadje, D. (2024). The implications of pesticide residue in food crops on human health: A critical review. Discov. Agric. 2, 123. doi: 10.1007/s44279-024-00141-z.

[S42] Jia, Q., Liao, G., Chen, L., Qian, Y., Yan, X., and Qiu, J. (2024). Pesticide residues in animal-derived food: Current state and perspectives. Food Chem. 438, 137974. doi: 10.1016/j.foodchem.2023.137974.

[S43] Pathak, V.M., Verma, V.K., Rawat, B.S., Kaur, B., Babu, N., Sharma, A., Dewali, S., Yadav, M., Kumari, R., Singh, S., et al. (2022). Current status of pesticide effects on environment, human health and its eco-friendly management as bioremediation: A comprehensive review. Front. Microbiol. 13, 962619. doi: 10.3389/fmicb.2022.962619.

[S44] Stenberg, J.A., Sundh, I., Becher, P.G., Björkman, C., Dubey, M., Egan, P.A., Friberg, H., Gil, J.F., Jensen, D.F., Jonsson, M., et al. (2021). When is it biological control? A framework of definitions, mechanisms, and classifications. J. Pest Sci. 94, 665–676. doi: 10.1007/s10340-021-01354-7.

[S45] Tyagi, A., Lama Tamang, T., Kashtoh, H., Mir, R.A., Mir, Z.A., Manzoor, S., Manzar, N., Gani, G., Vishwakarma, S.K., Almalki, M.A., et al. (2024). A review on biocontrol agents as a sustainable approach for crop disease management: Applications, production, and future perspectives. Horticulturae 10, 805. doi: 10.3390/horticulturae10080805.

[S46] Nchu, F. (2024). Sustainable biological control of pests: The way forward. Appl. Sci. 14, 2669. doi: 10.3390/app14072669.

[S47] Lee, J., Kim, S., Jung, H., Koo, B.-K., Han, J.A., and Lee, H.-S. (2023). Exploiting bacterial genera as biocontrol agents: Mechanisms, interactions and applications in sustainable agriculture. J. Plant Biol. 66, 485–498. doi: 10.1007/s12374-023-09404-6.

[S48] Lahlali, R., Ezrari, S., Radouane, N., Kenfaoui, J., Esmaeel, Q., El Hamss, H., Belabess, Z., and Barka, E.A. (2022). Biological control of plant pathogens: A global perspective. Microorganisms 10, 596. doi: 10.3390/microorganisms10030596.

[S49] Beyari, E.A. (2025). Alternatives to chemical pesticides: The role of microbial biocontrol agents in phytopathogen management: A comprehensive review. J. Plant Pathol. 107, 291–314. doi: 10.1007/s42161-024-01808-8.

[S50] Sharma, P., and Gaur, N. (2021). Microbial biopesticides use in insect-pest management: An overview. In Microbial Biotechnology in Crop Protection, M. Kaushal and R. Prasad, eds. (Springer Singapore), pp. 123–145. doi: 10.1007/978-981-16-0049-4_5.

[S51] Shahwar, D., Mushtaq, Z., Mushtaq, H., Alqarawi, A.A., Park, Y., Alshahrani, T.S., and Faizan, S. (2023). Role of microbial inoculants as biofertilizers for improving crop productivity: A review. Heliyon 9, e16134. doi: 10.1016/j.heliyon.2023.e16134.

[S52] Colmenarez, Y.C., and Vasquez, C. (2024). Benefits associated with the implementation of biological control programmes in Latin America. BioControl 69, 303–320. doi: 10.1007/s10526-024-10260-7.

[S53] Fleischer, S.J., Blom, P.E., and Weisz, R. (1999). Sampling in precision IPM: When the objective is a map. Phytopathology 89, 1112–1118. doi: 10.1094/PHYTO.1999.89.11.1112.

[S54] Deguine, J.-P., Aubertot, J.-N., Flor, R.J., Lescourret, F., Wyckhuys, K.A.G., and Ratnadass, A. (2021). Integrated pest management: Good intentions, hard realities. A review. Agron. Sustain. Dev. 41, 38. doi: 10.1007/s13593-021-00689-w.

[S55] Angon, P.B., Mondal, S., Jahan, I., Datto, M., Antu, U.B., Ayshi, F.J., and Islam, M.S. (2023). Integrated pest management (IPM) in agriculture and its role in maintaining ecological balance and biodiversity. Adv. Agric. 2023, 5546373. doi: 10.1155/2023/5546373.

[S56] Tamò, M., Glitho, I., Tepa-Yotto, G., and Muniappan, R. (2022). How does IPM 3.0 look like (and why do we need it in Africa)? Curr. Opin. Insect Sci. 53, 100961. doi: 10.1016/j.cois.2022.100961.

[S57] Lane, D. (2025). Comparing integrated pest management approaches: European Union versus United States. J. Integr. Pest Manag. 16, 21. doi: 10.1093/jipm/pmaf021.

[S58] Zhou, W., Arcot, Y., Medina, R.F., Bernal, J., Cisneros-Zevallos, L., and Akbulut, M.E.S. (2024). Integrated pest management: An update on the sustainability approach to crop protection. ACS Omega 9, 41130–41147. doi: 10.1021/acsomega.4c06628.

[S59] Love, M., Magarey, R.D., Holderman, B.L., Carley, D.S., Maggi, F., and Singer, N. (2025). Tracking sustainability in crop pest management in the United States using an eco-efficiency index. Front. Insect Sci. 5, 1582496. doi: 10.3389/finsc.2025.1582496.

[S60] Pawar, A.R., Kumari, S., Sootrakar, K., Hiremath, S., Singh, N., Awasthi, A., Dinesh, K.H.S., and Thiruvengadam, K. (2024). Advancing integrated pest management: Strategies for minimizing pesticide use. Int. J. Res. Agron. 7, 960–964. doi: 10.33545/2618060X.2024.v7.i12l.2359.

[S61] Treiber, M., Theunissen, T., Grebner, S., Witting, J., and Bernhardt, H. (2023). How to Successfully Orchestrate Content for Digital Agriecosystems. Agriculture 13, 1003. doi: 10.3390/agriculture13051003.

[S62] Russo, I., Vecchio, R., Viscecchia, R., Cembalo, L., and De Devitiis, B. (2025). Factors affecting farmers' adoption of sustainable pest management practices: A scoping review. Environ. Dev. Sustain. Published online October 27, 2025. doi: 10.1007/s10668-025-06937-3.

[S63] Mulugeta, T., Ilomo, M., Mueke, A., Onyango, C., Matsaunyane, L., Kritzinger, Q., and Alexandersson, E. (2024). Smallholder farmers' knowledge, attitudes, and practices (KAP) regarding agricultural inputs with a focus on agricultural biologicals. Heliyon 10, e26719. doi: 10.1016/j.heliyon.2024.e26719.

[S64] Mweke, A., Alexandersson, E., Mulugeta, T., Ilomo, M., Kritzinger, Q., Matsuanyane, L., and Onyango, C.M. (2025). Knowledge, attitude, and practice (KAP) analysis of agricultural biologicals among smallholder farmers across three counties in Kenya. J. Agric. Food Res. 19, 101614. doi: 10.1016/j.jafr.2024.101614.

[S65] Forouzani, M., Bondori, A., and Mombini, A.S. (2024). Why are farmers reluctant to accept biological inputs? A structural equation model of technology adoption. Front. Sustain. Food Syst. 8, 1360887. doi: 10.3389/fsufs.2024.1360887.

[S66] Kule, E.K., Obia, A., Agole, D., Okello, D.M., and Odongo, W. (2025). Farmer perceptions and their implications for adoption of sustainable agricultural intensification practices. Discov. Sustain. 6, 210. doi: 10.1007/s43621-025-00929-z.

[S67] Shahidullah, A.K.M., Islam, A., and Rahman, M. (2023). Knowledge, attitude, and practice of pesticide use by vegetable growers in Bangladesh: A health literacy perspective in relation to non-communicable diseases. Front. Sustain. Food Syst. 7, 1199871. doi: 10.3389/fsufs.2023.1199871.

[S68] Sarrocco, S. (2023). Biological Disease Control by Beneficial (Micro)Organisms: Selected Breakthroughs in the Past 50 Years. Phytopathology 113, 732–740. doi: 10.1094/PHYTO-11-22-0405-KD.

[S69] Zaidan, E., Al-Saidi, M., and Hammad, S.H. (2019). Sustainable development in the Arab world–Is the Gulf Cooperation Council (GCC) region fit for the challenge? Dev. Pract. 29, 670–681. doi: 10.1080/09614524.2019.1628922.
